# Lychee Seed Extract Targets Proliferation, Differentiation, and Cell Cycle Proteins to Suppress Human Colorectal Tumor Growth in Xenograft Models

**DOI:** 10.3390/ijms26199786

**Published:** 2025-10-08

**Authors:** Szu-Nian Yang, Yi-Ping Chang, Oscar C. Y. Yang, Chi-Sheng Wu, Chiu-Chen Huang, Jia-Feng Chang, Chia-Ming Liang, Shun-Tai Dai, Lung Chen, Chih-Ping Hsu

**Affiliations:** 1 Department of Psychiatry, Taipei Veterans General Hospital, Taoyuan Branch, Taoyuan 330023, Taiwan; ysn56725@ms4.hinet.net; 2 Department of Oral Hygiene, Jen-Teh Junior College of Medicine, Nursing and Management, Miaoli 356006, Taiwan; 3 Division of Nephrology, Department of Internal Medicine, Taipei Veterans General Hospital, Taoyuan Branch, Taoyuan 330023, Taiwan; D228@tyvh.gov.tw; 4 Department of Healthcare Information and Management, Taoyuan Campus, Ming Chuan University, Taoyuan 333321, Taiwan; 5 Division of Hematology and Oncology, Department of Internal Medicine, Taipei Veterans General Hospital, Taoyuan Branch, Taoyuan 330023, Taiwan; yanch856@gmail.com; 6 Renal Care Research and Health Promotion Association, New Taipei City 220050, Taiwan; luke.chisheng@gmail.com; 7 Department of Post-Baccalaureate Veterinary Medicine, Asia University, Taichung 413305, Taiwan; cch99@asia.edu.tw; 8 Department of Nursing, Yuanpei University of Medical Technology, Hsinchu 300102, Taiwan; 9 Division of General Surgery, Department of Surgery, Taipei Veterans General Hospital, Taoyuan Branch, Taoyuan 330023, Taiwan; D458@tyvh.gov.tw; 10 Department of Medical Laboratory Science and Biotechnology, Yuanpei University of Medical Technology, Hsinchu 300150, Taiwan; daishuntai@yahoo.com.tw (S.-T.D.); chenlun9815@gmail.com (L.C.)

**Keywords:** colorectal cancer, lychee seed extract, EGFR, p21, p53, Ki-67, xenograft model, chemoprevention

## Abstract

Colorectal cancer (CRC) remains a leading global health challenge, and natural products are increasingly explored for their multi-targeted therapeutic potential. *Litchi chinensis* seed extract (LCSE) has shown promising anti-cancer activity in vitro, though its in vivo effects remain underexplored. LCSE was analyzed by colorimetric assays and HPLC to quantify the phytochemical composition. Nude mice bearing HT-29 or SW480 xenografts were orally administered LCSE (0.1 or 0.6 g/kg) daily for 14 days. Tumor volume was measured, and immunohistochemistry was used to assess EGFR, p21, p53, Ki-67, CEA, CK20, CDX2, and Bax expression. Phytochemical profiling demonstrated LCSE contains abundant phenolics and flavonoids, with gallic acid as a predominant constituent, underscoring the potential bioactive properties. LCSE significantly inhibited tumor growth in HT-29 xenografts and dose-dependently reduced EGFR, p21, p53, cell cycle proteins and proliferation/differentiation markers. In SW480 tumors, inhibitory effects were evident primarily at the higher dose, with limited reduction in p53 expression. Bax levels remained unchanged in both models, indicating a non-apoptotic mechanism. No systemic toxicity was observed in treated mice. LCSE exhibits dose-dependent anti-tumor activity in CRC xenografts, likely mediated through suppression of proliferation and modulation of key regulatory proteins rather than apoptosis. These findings support LCSE as a safe, multi-target botanical candidate for CRC intervention and justify further mechanistic and translational studies.

## 1. Introduction

Colorectal cancer (CRC) is one of the leading causes of cancer-related morbidity and mortality worldwide, with over 1.9 million new cases and 935,000 deaths estimated annually. Despite advances in surgical techniques, chemotherapeutic regimens (such as FOLFOX and FOLFIRI), and targeted therapies (e.g., anti-EGFR and anti-VEGF agents), clinical outcomes remain limited by treatment resistance, systemic toxicity, and disease recurrence [1,2,3]. Therefore, there is an urgent need for safer, multi-targeted, and more tolerable treatment strategies for CRC patients.

The progression of CRC is driven by the dysregulation of key cellular processes including proliferation, apoptosis, and differentiation, often mediated through aberrant signaling pathways. Among these, epidermal growth factor receptor (EGFR) is frequently overexpressed and promotes tumor growth via activation of the RAS/RAF/MEK/ERK and PI3K/AKT cascades [4]. The tumor suppressor p53, along with its downstream effector p21, plays a central role in regulating cell cycle arrest and apoptosis, and both are commonly altered in CRC [5,6]. Additional molecular markers such as Ki-67 (proliferation), CEA (tumor burden), CK20, and CDX2 (epithelial differentiation) are widely used to evaluate tumor progression and prognosis in CRC [7,8,9].

In recent years, natural products have attracted growing interest as sources of anti-cancer agents due to their pleiotropic effects and generally favorable safety profiles. Lychee (*Litchi chinensis*) seed extract (LCSE), traditionally used in Chinese medicine, is rich in polyphenolic compounds such as epicatechin, catechin, and procyanidins, which have been shown to possess antioxidant, anti-inflammatory, and anti-proliferative activities [10,11,12]. In vitro studies have suggested that LCSE may suppress cancer cell growth by modulating pathways such as NF-κB and MAPK, promoting cell cycle arrest, and inducing apoptosis [13].

Polyphenols, including those found in LCSE, have been demonstrated to interfere with multiple tumorigenic pathways. For example, lychee-derived procyanidins inhibit epithelial–mesenchymal transition and trigger apoptosis via Akt/GSK-3β signaling in prostate cancer cells [14], while dietary polyphenols have been proposed as promising adjuvants in CRC therapy by modulating immune responses and inhibiting tumor-promoting inflammation [15]. These findings suggest that LCSE, as a polyphenol-rich botanical, may exert chemopreventive effects in CRC through coordinated modulation of oncogenic and tumor suppressor pathways [14,15,16]. However, most existing evidence is limited to in vitro models, and in vivo validation remains scarce.

To address this gap, we investigated the anti-tumor effects of LCSE using two well-characterized human CRC xenograft models—HT-29, which displays a moderately differentiated epithelial phenotype and elevated EGFR expression, and SW480, a more aggressive, mesenchymal-type cell line harboring mutant p53. Our objectives were to: (1) evaluate the safety of LCSE in vivo, (2) assess its ability to suppress tumor growth, and (3) analyze its effects on key molecular markers involved in cell cycle regulation, proliferation, and differentiation.

By elucidating the molecular effects of LCSE in these xenograft models, this study aims to support its development as a potential multi-target botanical candidate for integrative CRC therapy.

## 2. Results

### 2.1. Phytochemical Characterization of Litchi chinensis Seed Extract

**Figure 1:**
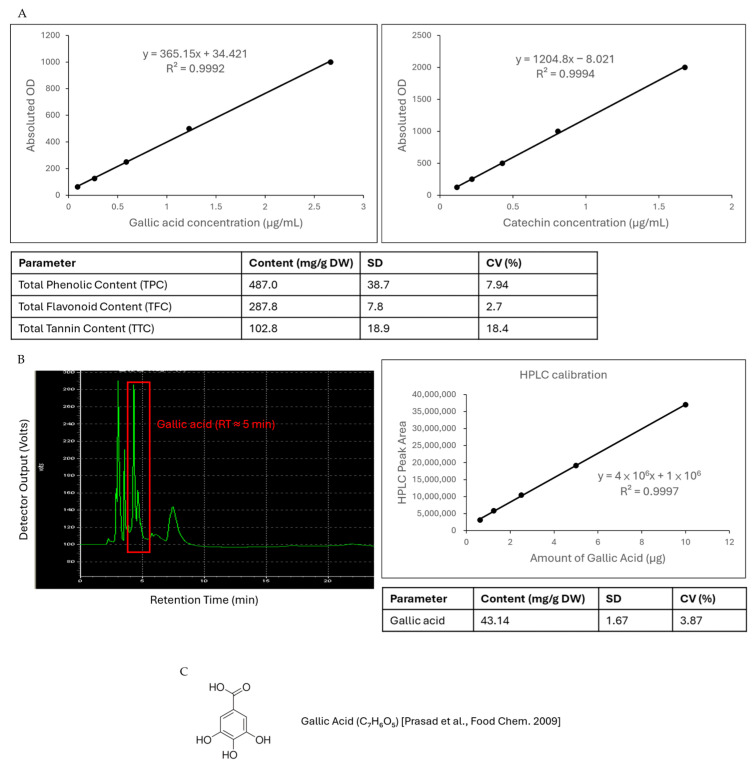
Quantification and profiling of phenolic compounds in *Litchi chinensis* seed extract (LCSE). (**A**) Calibration curves for gallic acid and catechin used for determination of total phenolic content (TPC), total flavonoid content (TFC), and total tannin content (TTC). The bar table summarizes the mean ± SD values (mg/g DW) and coefficients of variation (CV%). (**B**) Representative HPLC chromatogram of LCSE showing a prominent peak at retention time (RT) ≈ 5 min, identified as gallic acid by external standard comparison. The right panel shows the linear calibration curve for gallic acid quantification (R^2^ = 0.9997) and the corresponding content of gallic acid (mg/g DW). (**C**) Chemical structure of gallic acid (C_7_H_6_O_5_), adapted from Prasad et al. [18].

Colorimetric assays demonstrated that LCSE contains abundant phenolic compounds, with a total phenolic content (TPC) of 487.0 ± 38.7 mg GAE/g DW, a total flavonoid content (TFC) of 287.8 ± 7.8 mg QE/g DW, and a total tannin content (TTC) of 102.8 ± 18.9 mg TAE/g DW (Figure 1A). These findings confirm that LCSE is a polyphenol-rich extract and are consistent with previous reports describing high levels of phenolics in lychee seeds and pericarps [17,18].

HPLC profiling further characterized the phenolic composition of LCSE. Using gallic acid as an external standard, an excellent linear calibration curve was obtained (y = 4 × 10^6^ x + 1 × 10^6^, R^2^ = 0.9997), confirming a strong correlation between peak area and concentration (Figure 1B). A distinct peak was detected at approximately 5 min retention time, corresponding to gallic acid, and its concentration in LCSE was calculated as 43.14 ± 1.67 mg/g DW (CV = 3.87%), which aligns with previously reported HPLC-based quantification of gallic acid in lychee seed extracts [19,20].

In addition, several high-intensity peaks were clustered around the 10 min retention time region, suggesting that the majority of LCSE’s phenolic constituents—such as procyanidins, catechin derivatives, and other flavonoids—elute within this window, which is in agreement with published chromatographic profiles of lychee seed polyphenols [20,21,22]. This observation reinforces earlier findings that procyanidins B2, B3, and other oligomeric flavan-3-ols are abundant in lychee seed preparations [21,23].

Collectively, these findings demonstrate that LCSE is rich in phenolic and flavonoid compounds, with gallic acid identified as a principal constituent (Figure 1C), thereby providing a robust biochemical foundation for further investigation of its antioxidant and anti-tumor activities. 

### 2.2. LCSE Exhibits No Overt Toxicity in Male and Female Mice

Although natural compounds are often perceived as safe, proper in vivo toxicity evaluation is critical to ensure their suitability for therapeutic use. Previous studies have suggested that LCSE contains polyphenolic constituents with potential bioactivity, but its safety profile remains to be fully clarified in long-term settings [24]. In this study, we assessed the general toxicity of LCSE in both male and female mice through body weight monitoring and serum hematological and biochemical analyses.

**Figure 2:**
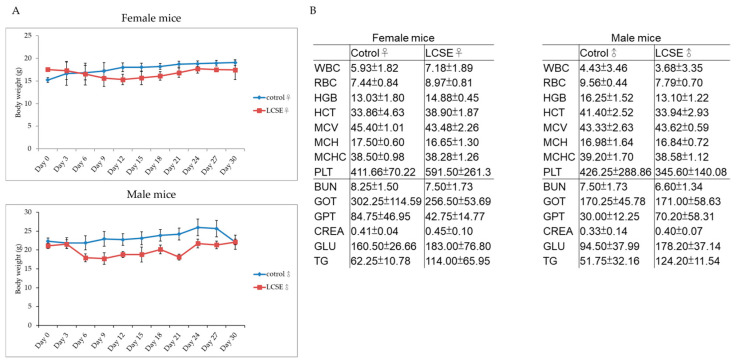
Body weight monitoring and blood analysis in male and female mice after 30-day oral administration of lychee seed extract (LCSE). (**A**) Body weight was recorded every 3 days. No significant weight loss was observed in either sex. (**B**) Hematological and biochemical parameters were measured at the end of treatment. All values remained within normal physiological ranges. Data are shown as mean ± SD. No significant differences were observed between LCSE and control groups.

Mice were orally administered LCSE daily for 30 days. As shown in Figure 2A, body weight progression in both sexes showed no significant differences between LCSE-treated and control groups throughout the study period. While minor fluctuations were observed, the overall trends remained stable. Comprehensive blood tests conducted on day 30 revealed no abnormalities in hematological parameters, including WBC, RBC, HGB, HCT, PLT, and related indices. Similarly, key biochemical markers—such as BUN, creatinine (CREA), liver enzymes (GOT, GPT), glucose (GLU), and triglycerides (TG)—remained within normal physiological ranges, with no signs of liver, kidney, or metabolic toxicity (Figure 2B). These findings confirm that LCSE is well tolerated at the tested dose in both male and female mice, with no overt systemic toxicity detected.

### 2.3. LCSE Inhibits Tumor Growth in a Xenograft Model of CRC

To evaluate the in vivo anti-tumor efficacy of LCSE, colorectal tumor cells were subcutaneously injected into the flanks of nude mice. Once the tumors reached approximately 100 mm^3^ in volume, the mice were orally administered with LCSE at two different doses (0.1 g/kg and 0.6 g/kg) daily for 14 consecutive days. Tumor volumes were measured on day 7 and day 14 of treatment.

**Figure 3:**
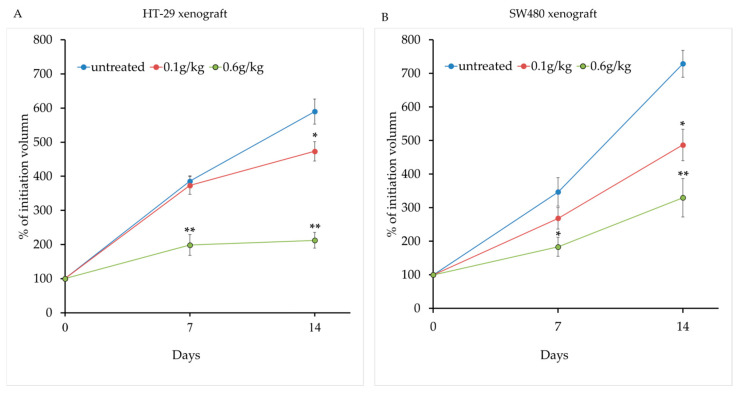
Lychee seed extract suppresses tumor growth in nude mice bearing colorectal cancer xenografts. (**A**) Tumor growth curves of mice treated with vehicle, 0.1 g/kg, or 0.6 g/kg lychee seed extract for 14 days, starting at ~100 mm^3^ tumor volume. Measurements were taken on days 7 and 14. (**B**) Tumor volumes on day 14. The 0.6 g/kg group showed significantly reduced tumor size compared to control. Data are shown as mean ± SEM. * *p* < 0.05, ** *p* < 0.01 vs. control.

As shown in Figure 3A, tumor volumes progressively increased in the control group, while both treatment groups exhibited slower tumor growth. Notably, mice receiving the high dose of LCSE (0.6 g/kg) showed a clear suppression of tumor expansion over time, with a statistically significant difference emerging by day 14. The low-dose group (0.1 g/kg) also displayed moderate tumor growth inhibition compared to the control group, though the effect was less pronounced. Quantitative comparison of tumor volumes on day 14 is summarized in Figure 3B. The high-dose group showed a significantly smaller tumor volume relative to controls (*p* < 0.01), while the low-dose group exhibited a trend toward reduction with statistical significance at the *p* < 0.05 level. These findings suggest a dose-dependent anti-tumor effect of LCSE in this xenograft model of CRC.

### 2.4. LCSE Modulates EGFR, p21, and p53 Expression in HT-29 CRC Xenografts

To visually assess the effects of LCSE on protein expression in vivo, immunohistochemical staining was performed on tumor sections from HT-29 CRC xenografts. Tumor-bearing nude mice were treated with vehicle, 0.1 g/kg, or 0.6 g/kg LCSE for 14 days, and tumor tissues were subjected to immunohistochemical analysis targeting three key CRC–associated proteins: EGFR, p21, and p53.

**Figure 4:**
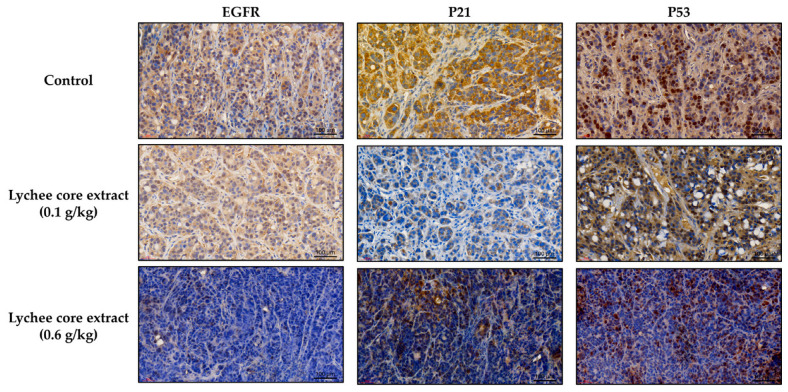
Immunohistochemical staining of EGFR (**left**), p21 (**middle**), and p53 (**right**) in HT-29 xenograft tumors after 14 days of treatment with lychee seed extract. Staining intensity decreased in a dose-dependent manner, with the 0.6 g/kg group showing the greatest reduction (Scale bar ≈ 100 µm).

Based on visual inspection of the stained tissue sections, tumors from the control group exhibited strong, widespread staining for all three proteins. EGFR showed intense brown staining along cell membranes and in the cytoplasm, p21 was moderately expressed in both the nucleus and cytoplasm, while p53 was prominently localized in the nucleus (Figure 4). In contrast, treatment with LCSE led to a visible, dose-dependent reduction in staining intensity for all three markers. The 0.6 g/kg group showed the most obvious decrease in staining, with EGFR expression appearing faint or patchy, and both p21 and p53 staining markedly diminished compared to the control. The reduction in staining suggests that LCSE effectively downregulates these proteins at the tumor tissue level, a finding further supported by the quantitative analysis described in the following section.

### 2.5. LCSE Reduces EGFR, p21, and p53 Expression in HT-29 and SW480 Xenograft Tumors

To further evaluate the protein-level effects of LCSE in vivo, we quantified immunohistochemical staining intensities of EGFR, p21, and p53 in two CRC xenograft models: HT-29 and SW480.

**Figure 5:**
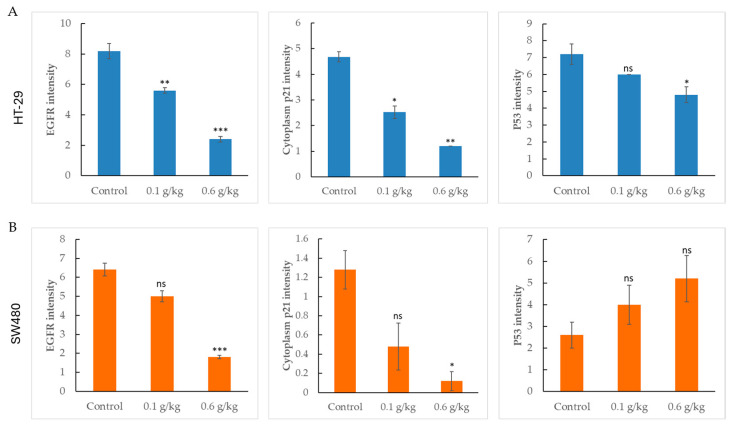
Quantification of EGFR, p21, and p53 expression in HT-29 and SW480 colorectal cancer xenograft tumors after lychee seed extract treatment. (**A**) In HT-29 xenografts, lychee seed extract significantly reduced expression of all three proteins in a dose-dependent manner. (**B**) In SW480 xenografts, EGFR and p21 were reduced mainly at the high dose (0.6 g/kg), while p53 expression remained unaffected. Data are shown as mean ± SEM. * *p* < 0.05, ** *p* < 0.01, *** *p* < 0.001 vs. control; ns, not significant.

In HT-29 tumors (Figure 5A), treatment with LCSE led to a clear, dose-dependent reduction in all three markers. EGFR and p21 showed significant decreases even at 0.1 g/kg, while the 0.6 g/kg group exhibited the most profound suppression (*p* < 0.01 to *p* < 0.001). p53 levels were also significantly reduced, consistent with the visual findings in tumor sections. In contrast, the response in SW480 xenografts (Figure 5B) displayed a weaker and more selective inhibition pattern. EGFR expression was only significantly reduced at the high dose. p21 showed a mild decrease, reaching significance at 0.6 g/kg, while p53 levels remained unchanged across all treatment groups.

This dual xenograft analysis reveals that LCSE consistently reduces EGFR and p21 expression in both models, but its impact on p53 is cell-type dependent, being effective in HT-29 but not in SW480 tumors. These results underscore the context-dependent molecular effects of LCSE in CRC.

### 2.6. Dose-Dependent Suppression of Proliferation and Differentiation Markers by LCSE in HT-29 and SW480 Xenografts

To explore the effects of LCSE on colorectal tumor characteristics, we quantified the expression of proliferation and differentiation markers, along with the apoptotic regulator Bax, in HT-29 and SW480 xenografts.

**Figure 6:**
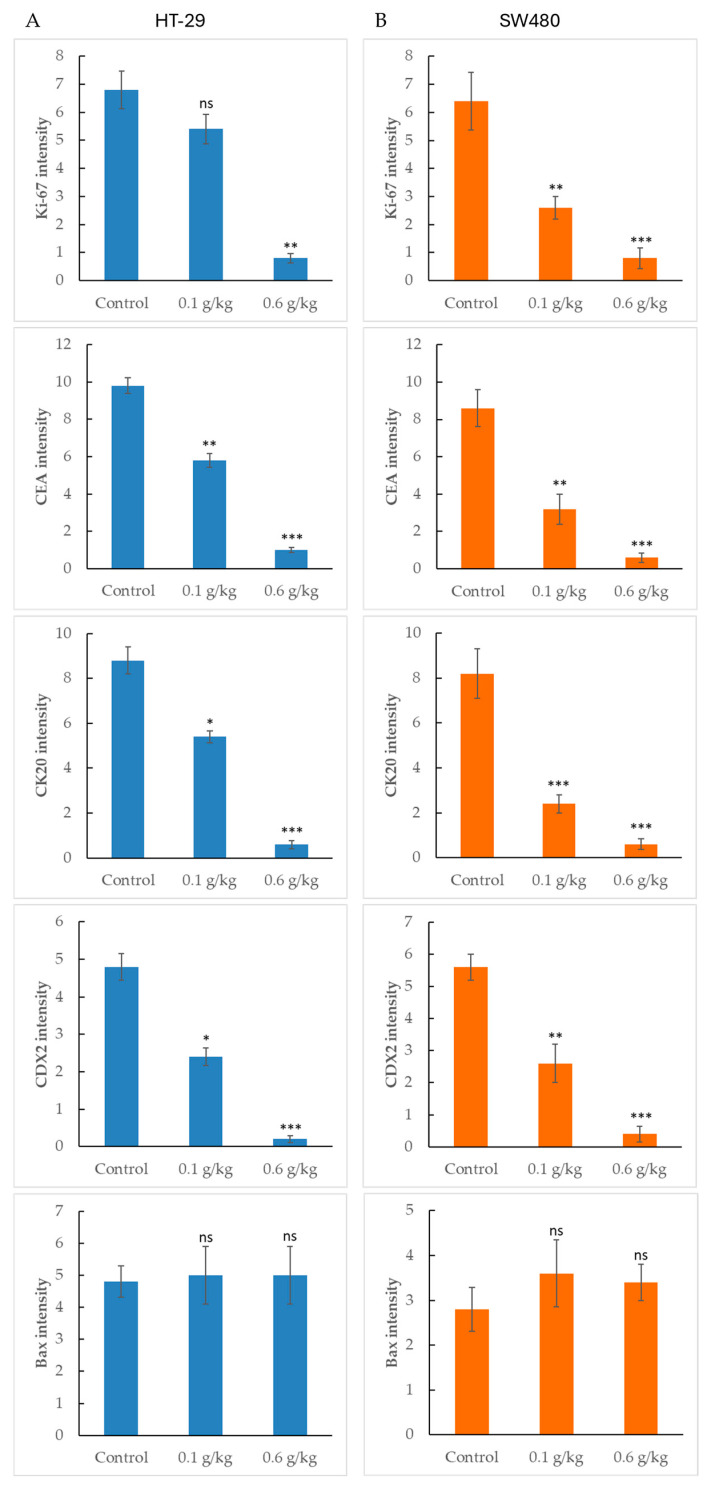
Quantification of Ki-67, CEA, CK20, CDX2, and Bax expression in HT-29 and SW480 xenograft tumors treated with lychee seed extract. (**A**) In HT-29 xenografts, lychee seed extract significantly reduced expression of Ki-67, CEA, CK20, and CDX2 in a dose-dependent manner, while Bax levels remained unchanged. (**B**) In SW480 xenografts, similar suppression of the four markers was observed, but Bax expression was not affected by treatment. Data are shown as mean ± SEM. * *p* < 0.05, ** *p* < 0.01, *** *p* < 0.001 vs. control; ns, not significant.

In HT-29 tumors (Figure 6A), Ki-67, CEA, CK20, and CDX2 showed significant dose-dependent reductions in staining intensity, particularly at 0.6 g/kg, consistent with suppression of tumor proliferation and epithelial lineage markers. SW480 tumors (Figure 6B) exhibited a similar trend with robust inhibition of all four markers at both doses, suggesting broad-spectrum effects on tumor identity and proliferation. Interestingly, despite these inhibitory effects, Bax expression remained unchanged across all treatment groups in both models. Neither low nor high-dose LCSE significantly affected Bax levels, as indicated by the lack of statistical difference from the control. This suggests that while LCSE modulates proliferative and differentiation pathways, it may not activate the intrinsic apoptotic pathway via Bax in these tumor models. The consistent suppression of tumor markers, coupled with stable Bax expression, highlights a selective regulatory mechanism that warrants further mechanistic investigation.

## 3. Discussion

Natural product-based therapies have gained increasing attention as adjuncts or alternatives to conventional chemotherapy in CRC, given their pleiotropic bioactivities and generally favorable safety profiles. LCSE, derived from *Litchi chinensis*, has been traditionally used in Asian medicine and is reported to contain high levels of polyphenols and flavonoids with anti-inflammatory and anti-cancer potential [24,25]. Our phytochemical characterization supports these reports, showing that LCSE is particularly rich in phenolic compounds, with high total phenolic and flavonoid contents and quantifiable levels of gallic acid (43.14 ± 1.67 mg/g DW) as a major constituent (Figure 1). Moreover, the HPLC chromatographic profile revealed that most phenolic constituents eluted around the 10-min retention window, suggesting that procyanidins, catechins, and other flavonoid derivatives account for a substantial proportion of the extract [17,18,19,20,21,22].

These findings provide a plausible biochemical rationale for the biological activities observed in subsequent experiments, as gallic acid and procyanidins have been reported to induce apoptosis, inhibit proliferation, and attenuate inflammatory signaling in CRC models [26,27]. Nevertheless, caution is warranted when extrapolating these results to clinical applications. The phytochemical composition of LCSE may vary depending on cultivar, harvest season, and extraction method, potentially affecting reproducibility and bioactivity. Furthermore, the bioavailability and metabolism of gallic acid and other polyphenols in vivo remain incompletely understood, which could limit their systemic efficacy [28]. Together, these data highlight both the promise and the challenges of developing LCSE as a natural product-based intervention for CRC and underscore the importance of correlating phytochemical profiles with standardized biological outcomes.

In this study, we systematically evaluated the anti-tumor potential and safety of LCSE in two human CRC xenograft models, HT-29 and SW480. Our findings demonstrated that LCSE is well tolerated in vivo, as shown by the absence of significant changes in body weight and normal hematological and biochemical parameters in both male and female mice (Figure 2). These results support the safe use of LCSE in preclinical settings and align with previous toxicity reports showing no adverse effects of LCSE even at high doses in rodents [12]. Notably, LCSE exhibited significant tumor-suppressive effects in the HT-29 model, and more moderate effects in SW480 xenografts (Figure 3). This differential response may be attributed to the intrinsic molecular heterogeneity of the cell lines. HT-29 is characterized by a differentiated epithelial phenotype and high EGFR activity, while SW480 displays a more mesenchymal and aggressive profile with mutant p53 and altered Wnt signaling [29,30]. These features likely contribute to the enhanced sensitivity of HT-29 tumors to LCSE treatment.

Immunohistochemical analyses provided mechanistic insights into LCSE activity. In HT-29 tumors, LCSE significantly reduced the expression of EGFR, p21, and p53 in a dose-dependent manner (Figure 5A), suggesting disruption of oncogenic signaling and cell cycle regulation. In contrast, SW480 tumors showed only mild reductions in EGFR and p21, and no change in p53 expression (Figure 5B). These observations imply that LCSE exerts more pronounced molecular effects in tumors with intact or partially functional p53 and EGFR pathways. Such context-specific modulation is consistent with previous studies showing polyphenol-induced downregulation of EGFR and cyclin-related proteins in various CRC models [31,32].

Furthermore, LCSE suppressed the expression of Ki-67, CEA, CK20, and CDX2 in both models (Figure 6), indicating inhibitory effects not only on proliferation but also on epithelial lineage maintenance and tumor differentiation. Although complete abrogation of these markers was not observed, the consistent downward trend reinforces the notion that LCSE interferes with multiple hallmarks of tumor progression. Interestingly, despite these effects, Bax expression remained unchanged in both models (Figure 6), suggesting that LCSE does not activate canonical Bax-mediated apoptosis. This finding aligns with reports that some botanical compounds, including lychee-derived procyanidins, exert anti-cancer effects through autophagy induction or non-apoptotic growth suppression [33].

However, the absence of Bax activation does not entirely exclude the possibility of apoptosis, as extrinsic (death receptor-mediated) pathways, such as Fas/FasL or caspase-8 activation, could still be involved [34,35]. The lack of extrinsic apoptotic marker evaluation represents a limitation of this study, and future investigations will incorporate these analyses to clarify whether LCSE induces apoptosis through non-mitochondrial mechanisms. In addition, while we mentioned the potential involvement of autophagy, we acknowledge that this suggestion cannot be substantiated without direct experimental evidence. Our study did not evaluate canonical autophagy markers such as LC3-II, Beclin-1, or p62. Therefore, autophagy should be considered a possible but unconfirmed mechanism, which requires future validation [36,37]. We have revised the text to clarify that this remains a hypothesis rather than a conclusion.

These results are encouraging in that LCSE demonstrates multi-target inhibitory activity without systemic toxicity. The dose-dependent suppression of both proliferation- and differentiation-associated proteins indicates that LCSE may impair tumor plasticity and stemness-like features, especially in epithelial-type tumors. Such properties are highly desirable in cancer therapeutics, as they may prevent tumor regrowth and resistance.

Nevertheless, several aspects warrant further investigation. While we observed clear changes in key protein markers, additional mechanistic studies are required to delineate the precise signaling pathways involved. Apoptosis, autophagy, senescence, and metabolic stress should all be explored using molecular assays such as caspase activity, LC3-II detection, and transcriptomic profiling [38]. Moreover, the extract’s complex composition necessitates bioassay-guided fractionation to identify its active constituents and confirm structure–activity relationships [39]. Although both models are useful, broader validation in additional CRC subtypes, including MSI-H and CMS3 tumors, would strengthen the translational relevance of our findings.

Importantly, future studies should also consider combining LCSE with conventional chemotherapeutics or immune checkpoint inhibitors, as polyphenols are known to modulate the tumor microenvironment and enhance treatment response [40,41]. The potential role of LCSE in modulating gut microbiota and systemic inflammation may also contribute to its anti-cancer effects, as proposed in emerging CRC studies [42].

Overall, this study demonstrates LCSE is a well-tolerated and biologically active botanical compound with promising anti-tumor effects in CRC xenografts. Comprehensive phytochemical profiling confirmed that LCSE is rich in polyphenols and flavonoids, with gallic acid as a major constituent and most phenolic compounds eluting around the 10-min retention window (Figure 1), in line with previous reports identifying procyanidins and catechin derivatives as abundant bioactive molecules in LCSE [17,18,19,20,21,22]. This polyphenol-rich composition provides a biochemical basis for its ability to suppress tumor growth and modulate key molecular markers.

LCSE downregulated oncogenic and differentiation-related markers, including EGFR, p21, p53, and Ki-67, without triggering canonical apoptotic cascades, suggesting alternative growth-inhibitory mechanisms such as cell cycle arrest, differentiation blockade, or modulation of tumor-associated signaling pathways. Given that gallic acid and procyanidins have been reported to interfere with EGFR signaling, NF-κB activation, and cell cycle control in CRC models [26,27,28], these constituents may contribute synergistically to the observed anti-tumor effects.

Collectively, our findings provide robust in vivo evidence supporting LCSE as a promising multi-target natural agent for integrative colorectal cancer therapy and justify further mechanistic investigations to delineate its precise molecular targets, bioavailability, and translational potential.

## 4. Materials and Methods

### 4.1. Preparation of LCSE

Lychee seeds were collected from the fruit of *Litchi chinensis* after consumption. The seeds were first washed thoroughly, frozen at −20 °C overnight, and then homogenized using a tissue homogenizer (pre-chilled) to obtain a fine powder. The powdered lychee seeds were subjected to ethanol extraction by soaking in a solution of 70% ethanol in water (*v*/*v*) at a ratio of 1:10 (*w*/*v*) under constant stirring at room temperature for 24 h. After extraction, the mixture was filtered through Whatman filter paper, and the filtrate was concentrated using a rotary vacuum evaporator to remove ethanol under reduced pressure. The remaining aqueous extract was subsequently freeze-dried to obtain a stable LCSE powder.

For in vitro and in vivo experiments, the lyophilized LCSE powder was reconstituted in dimethyl sulfoxide (DMSO) to prepare stock solutions. Working concentrations were calculated based on the dry weight of the extract and diluted appropriately for each experimental setup.

### 4.2. Phytochemical Characterization of LCSE

Colorimetric Quantification: The total phenolic content (TPC) of LCSE was determined by the Folin–Ciocalteu colorimetric method as described previously [17] with minor modifications. Briefly, 100 µL of LCSE solution was mixed with 500 µL of 10% Folin–Ciocalteu reagent and incubated for 5 min at room temperature. Subsequently, 400 µL of 7.5% Na_2_CO_3_ solution was added, and the mixture was incubated for 30 min in the dark. Absorbance was measured at 765 nm using a microplate spectrophotometer, and TPC was expressed as mg gallic acid equivalents per g dry weight (mg GAE/g DW) based on a gallic acid calibration curve (0–200 µg/mL, R^2^ = 0.9997).

The total flavonoid content (TFC) was measured using the aluminum chloride colorimetric assay [17]. Briefly, 250 µL of sample was mixed with 1.25 mL distilled water and 75 µL of 5% NaNO_2_, allowed to react for 6 min, followed by addition of 150 µL of 10% AlCl_3_. After 5 min, 500 µL of 1 M NaOH was added and absorbance was recorded at 510 nm. TFC was calculated from a catechin standard curve and expressed as mg catechin equivalents per g DW (mg CE/g DW).

Total tannin content (TTC) was determined by the vanillin-HCl method [19]. Results were expressed as mg tannic acid equivalents per g DW (mg TAE/g DW).

HPLC Analysis of Gallic Acid: Quantitative determination of gallic acid in LCSE was performed by high-performance liquid chromatography (HPLC) using an Agilent 1260 Infinity system equipped with a C18 column (4.6 mm × 250 mm, 5 µm). The mobile phase consisted of solvent A (0.1% formic acid in water) and solvent B (acetonitrile) with a gradient elution program: 0–2 min, 5% B; 2–12 min, 5–20% B; 12–15 min, 20–25% B; flow rate 1.0 mL/min, detection at 280 nm. External calibration was performed using gallic acid standards (10–200 µg/mL), and peak identity was confirmed by comparing retention time (RT ≈ 5 min) with the reference standard. Quantitative results were expressed as mg gallic acid per g DW (mg/g DW).

### 4.3. Cell Culture of HT-29 and SW480

The human CRC cell lines HT-29 (ATCC^®^ HTB-38™) and SW480 (ATCC^®^ CCL-228™) were obtained from the Bioresource Collection and Research Center (BCRC, Hsinchu, Taiwan).

HT-29 cells were cultured in Dulbecco’s Modified Eagle’s Medium (DMEM; high glucose, 4.5 g/L; Gibco, Waltham, MA, USA) supplemented with 10% fetal bovine serum (FBS; Gibco, Waltham, MA, USA), 100 U/mL penicillin, and 100 µg/mL streptomycin (Gibco, Waltham, MA, USA). Cells were maintained at 37 °C in a humidified incubator with 5% CO_2_, and passaged every 2–3 days upon reaching 70–80% confluency using 0.25% trypsin-EDTA (Gibco, Waltham, MA, USA). SW480 cells were maintained in Leibovitz’s L-15 medium (Gibco, Waltham, MA, USA) supplemented with 10% FBS. According to the culture recommendation for this cell line, they were incubated at 37 °C in a CO_2_-free incubator (without CO_2_ supplementation), following ATCC guidelines for this cell line.

For tumor induction, both cell lines were harvested during logarithmic growth, washed, and resuspended in sterile phosphate-buffered saline (PBS) mixed with Matrigel (1:1 *v*/*v*; Corning, Corning, NY, USA) prior to subcutaneous injection into immunodeficient mice.

### 4.4. Tumor Xenograft Establishment in Nude Mice

Male and female nude mice (BALB/c nude, 5–6 weeks old) were purchased from the National Laboratory Animal Center (NLAC, Taipei, Taiwan). For safety evaluation, mice were randomly assigned to receive daily oral administration of LCSE at the indicated dose or water (control group) for 30 days. Body weights were measured every 3 days, and animals were observed continuously for any clinical signs or behavioral abnormalities. On day 30, mice were sacrificed for blood collection; hematological and serum biochemical parameters were assessed to evaluate systemic safety.

For tumor xenograft assays, HT-29 or SW480 cells grown to logarithmic phase were harvested, counted, and resuspended at 5 × 10^6^ cells per 100 µL in sterile PBS mixed 1:1 with Matrigel. The cell suspension was injected subcutaneously into the flank of nude mice. When tumors reached an approximate volume of ~100 mm^3^, daily oral administration of LCSE or water control commenced. Each experimental group consisted of 5 mice (n = 5). Tumor growth was monitored by measuring two perpendicular diameters (length and width) with a digital vernier caliper (Mitutoyo, Kawasaki, Japan) every 3–4 days, and tumor volume was calculated using the standard formula: V = (length × width^2^)/2. Tumor volume was measured on days 7 and 14 post-treatment, and mice were sacrificed thereafter. Tumors were excised, measured for weight and volume, and processed for histological analyses. This protocol follows established xenograft methodologies in CRC models, where subcutaneous injection of 5 × 10^6^ cells and serial tumor measurements are standard practice for evaluating natural compound efficacy in vivo [43]. All animal procedures were approved by the Institutional Animal Care and Use Committee (IACUC) of Asia University, under approval number 112-asia-02.

### 4.5. Immunohistochemistry Analysis

Tumor tissues excised from xenografted mice were fixed in 10% neutral-buffered formalin for 24 h, embedded in paraffin, and sectioned at a thickness of 4 μm. The sections were deparaffinized with xylene, rehydrated through a graded ethanol series, and subjected to heat-induced antigen retrieval using citrate buffer (pH 6.0) in a microwave oven. To block endogenous peroxidase activity, tissue sections were treated with 3% hydrogen peroxide for 10 min at room temperature. After rinsing with phosphate-buffered saline (PBS), the sections were incubated overnight at 4 °C with primary antibodies specific for EGFR, p21, p53, Ki-67, CEA, CK20, CDX2, and Bax. These antibodies, obtained from Cell Signaling Technology (Danvers, MA, USA) or Abcam (Cambridge, UK), were diluted according to the manufacturers’ protocols. Following incubation with primary antibodies, the sections were treated with a biotinylated secondary antibody and then with streptavidin–horseradish peroxidase conjugate using the Dako REAL™ EnVision Detection System (Agilent Technologies, Santa Clara, CA, USA). Immunoreactivity was visualized by chromogenic development with 3,3′-diaminobenzidine (DAB) substrate, and counterstaining was performed with hematoxylin. Finally, the slides were dehydrated, mounted, and examined under a light microscope. Staining intensity and the percentage of positively stained cells were analyzed using ImageJ software (version 1.54f, 2023; National Institutes of Health, Bethesda, MD, USA) to provide semi-quantitative evaluation of protein expression.

A limitation of the present study is the absence of pharmacokinetic/pharmacodynamic (PK/PD) evaluation to establish the optimal dosing strategy for LCSE. Although the doses selected were well tolerated and demonstrated anti-tumor activity in vivo, future studies incorporating PK/PD modeling and bioavailability analysis will be critical to define therapeutic windows and support clinical translation. Specifically, we note that subsequent studies should include PK/PD profiling, bioactive compound identification, and metabolism analysis to support translational development of LCSE.

### 4.6. Statistical Analysis

Data are expressed as mean ± SEM. Statistical significance was determined using unpaired two-tailed Student’s *t*-test or one-way ANOVA followed by Tukey’s post hoc test, as appropriate. A *p* value < 0.05 was considered statistically significant. All analyses were performed using GraphPad Prism 9.0 (GraphPad Software, San Diego, CA, USA). IHC quantification was conducted using ImageJ software.

## 5. Conclusions

LCSE significantly inhibited CRC xenograft growth, downregulated key oncogenic and differentiation markers (EGFR, p53, p21, Ki-67, CEA, CK20, CDX2), and exhibited low systemic toxicity, supporting its favorable safety profile. Phytochemical profiling confirmed its polyphenol-rich composition, with gallic acid as a principal constituent, providing a biochemical basis for its bioactivity. Our results indicate LCSE exerts anti-tumor effects through non-apoptotic pathways such as cell cycle regulation or differentiation blockade, potentially mediated by gallic acid and procyanidins. Collectively, our findings highlight LCSE as a promising, well-tolerated multi-target natural candidate for integrative CRC therapy and warrant further mechanistic and translational studies.

## Data Availability

All data used to support the findings of this study are available from the corresponding author, Chang, J.-F., upon reasonable request.
